# Cardiovascular Agents Affect the Tone of Pulmonary Arteries and Veins in Precision-Cut Lung Slices

**DOI:** 10.1371/journal.pone.0029698

**Published:** 2011-12-27

**Authors:** Annette D. Rieg, Rolf Rossaint, Stefan Uhlig, Christian Martin

**Affiliations:** 1 Department of Anaesthesiology, Institute of Pharmacology and Toxicology, Medical Faculty Aachen, RWTH-Aachen, Aachen, Germany; 2 Institute of Pharmacology and Toxicology, Medical Faculty Aachen, RWTH-Aachen, Aachen, Germany; McMaster University, Canada

## Abstract

**Introduction:**

Cardiovascular agents are pivotal in the therapy of heart failure. Apart from their action on ventricular contractility and systemic afterload, they affect pulmonary arteries and veins. Although these effects are crucial in heart failure with coexisting pulmonary hypertension or lung oedema, they are poorly defined, especially in pulmonary veins. Therefore, we investigated the pulmonary vascular effects of adrenoceptor agonists, vasopressin and angiotensin II in the model of precision-cut lung slices that allows simultaneous studies of pulmonary arteries and veins.

**Materials and Methods:**

Precision-cut lung slices were prepared from guinea pigs and imaged by videomicroscopy. Concentration-response curves of cardiovascular drugs were analysed in pulmonary arteries and veins.

**Results:**

Pulmonary veins responded stronger than arteries to α_1_-agonists (contraction) and β_2_-agonists (relaxation). Notably, inhibition of β_2_-adrenoceptors unmasked the α_1_-mimetic effect of norepinephrine and epinephrine in pulmonary veins. Vasopressin and angiotensin II contracted pulmonary veins via V_1a_ and AT_1_ receptors, respectively, without affecting pulmonary arteries.

**Discussion:**

Vasopressin and (nor)epinephrine in combination with β_2_-inhibition caused pulmonary venoconstriction. If applicable in humans, these treatments would enhance capillary hydrostatic pressures and lung oedema, suggesting their cautious use in left heart failure. Vice versa, the prevention of pulmonary venoconstriction by AT_1_ receptor antagonists might contribute to their beneficial effects seen in left heart failure. Further, α_1_-mimetic agents might exacerbate pulmonary hypertension and right ventricular failure by contracting pulmonary arteries, whereas vasopressin might not.

## Introduction

Treatment of acute and chronic heart failure is based on the therapy with cardiovascular agents that aim at improved ventricular contractility, enhanced coronary perfusion and reduced myocardial oxygen consumption. Importantly however, cardiovascular agents interact with the pulmonary vascular bed and thereby also influence myocardial function: First, contraction of pulmonary arteries (PAs) enhances right ventricular afterload and worsens right ventricular failure. Second, contraction of pulmonary veins (PVs) increases pulmonary capillary pressure and causes hydrostatic pulmonary oedema and deterioration of gas exchange. Thus, it is clinically important how PAs and PVs respond to cardiovascular agents. However, the differential effects of cardiovascular drugs along the pulmonary vascular bed are only incompletely defined. Most previous studies focused on PAs [1]–[5], probably due to their central role in pulmonary hypertension and right ventricular failure. Recently, PVs are receiving growing attention and their relevance in the regulation of total pulmonary vascular resistance is becoming evident [6]. Therefore, and due to completely different responses of PAs and PVs [7], simultaneous studies of both vessels are of great clinical interest; however, they are rare [4]. Further, pulmonary vessels differ from systemic vessels in their response to hypoxia, hypercapnia and acidosis [8], thus results from systemic vessels may not be applicable to the low pressure pulmonary vascular bed.

The aim of this study was to investigate the effects of adrenoceptor agonists, vasopressin and angiotensin II on PAs and PVs. We have chosen the model of precision-cut lung slices (PCLS), because it permits simultaneous studies of PAs and PVs. Further, guinea pigs (GPs) were chosen, because previous studies on airway pharmacology suggest that GPs may be a reasonable proxy of human lung tissue [9]. Our results indicate that GPs' PAs and PVs respond significantly different to adrenoceptor agonists, vasopressin and angiotension II. These findings suggest that differential effects of cardiovascular drugs along the pulmonary vascular tree might influence the success of heart failure therapy.

## Materials and Methods

### Guinea pigs (GPs)

Female Dunkin Hartley GPs (400±50 g) were obtained from Charles River (Sulzfeld, Germany) and held under standard conditions. All animal care and experimental procedures were performed according to the rules of the University Hospital Aachen (Aachen, Germany) and the Directive 2010/63/EU of the European Parliament. They were approved by the Landesamt für Natur, Umwelt und Verbraucherschutz Nordrhein-Westfalen (LANUV, approval-ID: 8.87–51.05.20.10.245).

### Precision-cut lung slices (PCLS)

PCLS from GPs (n = 39) were prepared as described before [9]. In brief, intraperitoneal anaesthesia was performed with 95 mg kg^−1^ pentobarbital (Narcoren; Garbsen, Germany) and its depth was monitored by missing reflexes. Afterwards, the abdomen was opened and the GP exsanguinated. Further, the trachea was cannulated, the diaphragm opened and the lungs filled with low melting point agarose (final concentration: 1.5%), containing 1 µM isoproterenol. To solidify the agarose, the lungs were covered with ice. The lobes were removed; tissue cores prepared and cut into 300 µm thick slices with a Krumdieck tissue slicer (Alabama Research & Development, Munford, AL, USA). Afterwards, PCLS were incubated at 37°C in a humid atmosphere in minimal essential medium (MEM), containing CaCl_2_ (1.8 mM), MgSO_4_ (0.8 mM), KCl (5.4 mM), NaCl (116.4 mM), glucose (16.7 mM), NaHCO_3_ (26.1 mM), Hepes (25.17 mM), sodium pyruvate, amino acids, vitamins and glutamine. To wash out the agarose from the slices, the MEM was changed every half hour during the first 2 h and every hour during the next 2 h. For overnight culture, MEM was completed with penicillin and streptomycin and changed every 24 h.

### Identification of the vessels, histology

**Figure 1:**
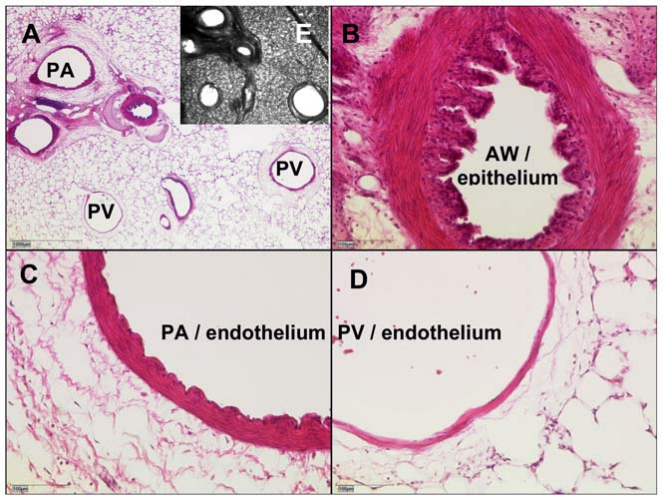
Identification of pulmonary vessels. **A**) A representative PCLS after staining with haematoxylin and eosin. **B**) epithelium of an airway (AW) **C**) Pulmonary artery (PA): endothelium with typical wrinkled inner lining and thick media **D**) Pulmonary vein (PV): endothelium without wrinkled inner lining and thin media **E**) corresponding unstained PCLS.

Pulmonary vessels were identified using the following criteria: PAs accompany the airways and PVs lie aside. After staining with haematoxylin and eosin (HE) PAs show a wrinkled inner lining and a thick media wall [7], as it is illustrated in Fig. 1A–D. Thus, after termination of the experiments PCLS were fixed in 4% formalin and embedded in paraffin. Sections (4 µM) were cut and HE-stained. Images were taken by a microscope (Leica, DM 600 B).

### Measurements and Imaging

At the beginning of the study, all agents were investigated with regard to the onset of their maximal contractile or relaxant effect. According to these results, the duration of exposure was defined for all agents, i.e. for (nor)epinephrine, isoproterenol, phenylephrine, procaterol, denopamine, CL 316243 and A 61603 3 minutes, for vasopressin 5 minutes and for angiotensin 10 minutes. The slices were exposed to different drugs on day one and two after preparation. Concentration-response curves were performed on pulmonary arteries and veins and cross sectional area of the vessels was calculated. Control experiments were performed on consecutive sections. Pulmonary vessels were imaged and digitised using a digital video camera (Leica Viscam 1280 or Leica DFC 280). The images were analysed with Optimas 6.5 (Media Cybernetics, Bothell, WA).

### Agents

All agents were purchased from Sigma-Aldrich (Steinheim, Germany), except CGP 20712 A, procaterol and CL 316243, which were from Tocris Bioscience (Ellisville, Missouri, USA).

### Statistics

Statistics was conducted using SAS software version 9.1 (SAS Institute, Cary, North Carolina, USA) and GraphPad Prism version 5.0 (GraphPad Software, La Jolla, USA). Homoscedasticity of values was evaluated. Changes of the vessel area are expressed as percentage of its initial area. All values are shown as mean ± SEM. Paired observations were analysed using the one sample t-test or the Wilcoxon signed rank test. Unpaired observations were compared using the Mann-Whitney Test. When the effect of increasing concentrations on the vessels was roughly linear, data were analysed using a linear mixed model analysis (LMM). In case of sigmoidal concentration-response curves; the standard logistic regression model was used to calculate and compare EC_50_ values. For LMM and logistic regression the AIC-criterion was used to select the preferred model. All p-values were adjusted for multiple comparisons by the false discovery rate [10]. P-values <0.05 were considered as significant. For all experiments, (n) indicates the numbers of animals.

## Results

### Stimulation of α- and β-receptors in PAs and PVs

The endogenous vasoactive compounds norepinephrine and epinephrine [(nor)epinephrine] represent cardiovascular agents, that are worldwide most commonly used to restore circulation in cardiac failure and shock.

**Figure 2:**
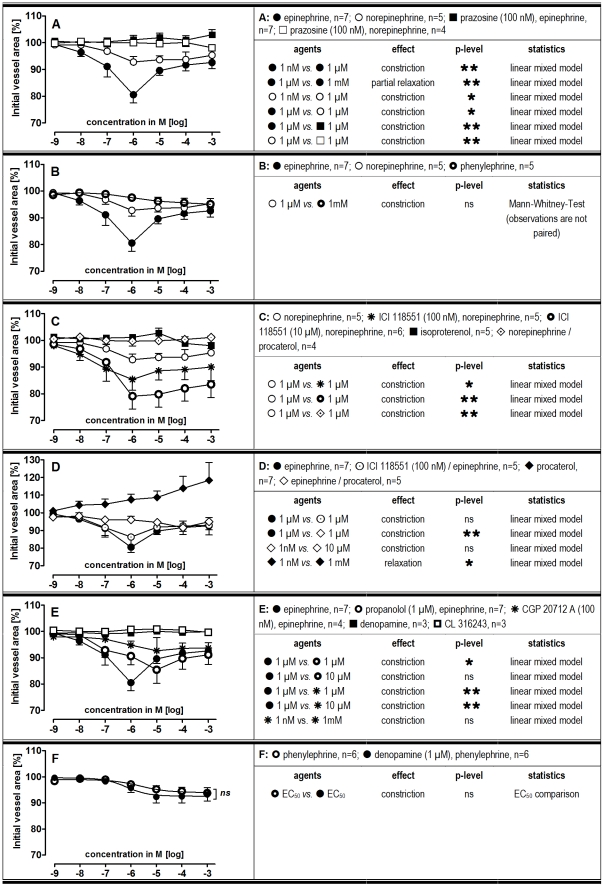
Responses of pulmonary arteries (PAs) to increasing dosages of adrenergic agents. **A-F)** Pre-treatment concentrations were fixed. P<0.05 are considered as statistical significant and are indicated as followed 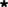 p<0.05, 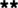 p<0.01 and 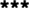 p<0.001.

In PAs, (nor)epinephrine induced maximal contraction at 1 µM, though epinephrine was more potent. Above 1 µM this difference disappeared, suggesting the additional activation of β_2_-adrenoceptors by epinephrine (Fig. 2A). Pre-treatment of PAs with the α_1_-antagonist prazosine (100 nM) prevented (nor)epinephrine-induced contraction (Fig. 2A). Comparable to norepinephrine, the α_1_-agonist phenylephrine induced only slight contraction in PAs (Fig. 2B). Similar results were obtained for the α_1_-agonist A 61603, whereas the α_2_-agonist clonidine had no effect (not shown). Pre-treatment of PAs with the selective β_2_-antagonist ICI 118551 (100 nM) enhanced norepinephrine-induced contraction and 10 µM ICI 118551 had an even stronger effect (Fig. 2C). However, ICI 118551 at 100 nM (Fig. 2D) or 10 µM (not shown) did not alter the effect of epinephrine. The β_1/2_-agonist isoproterenol had no effect in PAs (Fig. 2C), whereas the pure β_2_-agonist procaterol relaxed them (Fig. 2D). Further, procaterol completely abolished contraction due to (nor)epinephrine (Fig. 2C/D). Pre-treatment of PAs with the β_1/2_-antagonist propanolol (1 µM) shifted epinephrine-induced contraction rightwards, without altering its maximal effect (Fig. 2E). Pre-treatment with the β_1_-antagonist CGP 20712A (100 nM) abolished epinephrine-induced contraction (Fig. 2E). However, the β_1_-agonist denopamine had no effect on PAs. Further, denopamine given as pre-treatment prior to increasing concentrations of phenylephrine did not enhance phenylephrine-induced contraction (Fig. 2F). Also the β_3_-agonist CL 316243 had no effect (Fig. 2E). Finally, the tone of PAs was not affected by prazosine, CGP 20712A, ICI 118551 and propanolol (Table 1).

**Table 1:** Influence of various antagonists and inhibitors on the initial vessel area.

agents	PA mean (%)	n	SEM	p-value	PV mean (%)	n	SEM	p-value
**prazosine 100 nM**	102	8	3.7	0.64	103	8	3.2	0.33
**CGP 20712 A 100 nM**	101	5	1.3	0.81	102	5	1.5	0.31
**ICI 188551 10 µM**	101	6	1.2	1	95	9	5.5	0.34
**ICI 188551 100 nM**	100	10	1.2	0.7	97	10	3.5	0.38
**propanolol 1 µM**	97	8	1.2	0.06	93	9	4.7	0.16
**indomethacin 10 µM**	98	7	0.9	0.15	95	9	1.1	0.001
**SR 49059 10 nM**	102	3	3.2	1	101	3	1.4	1
**losartan 1 µM**	100	4	2.4	1	98	4	1.2	0.25

Observations were paired, thus statistics was conducted using the One sample t-test or the Wilcoxon Test. P<0.05 are considered as statistical significant.

**Figure 3:**
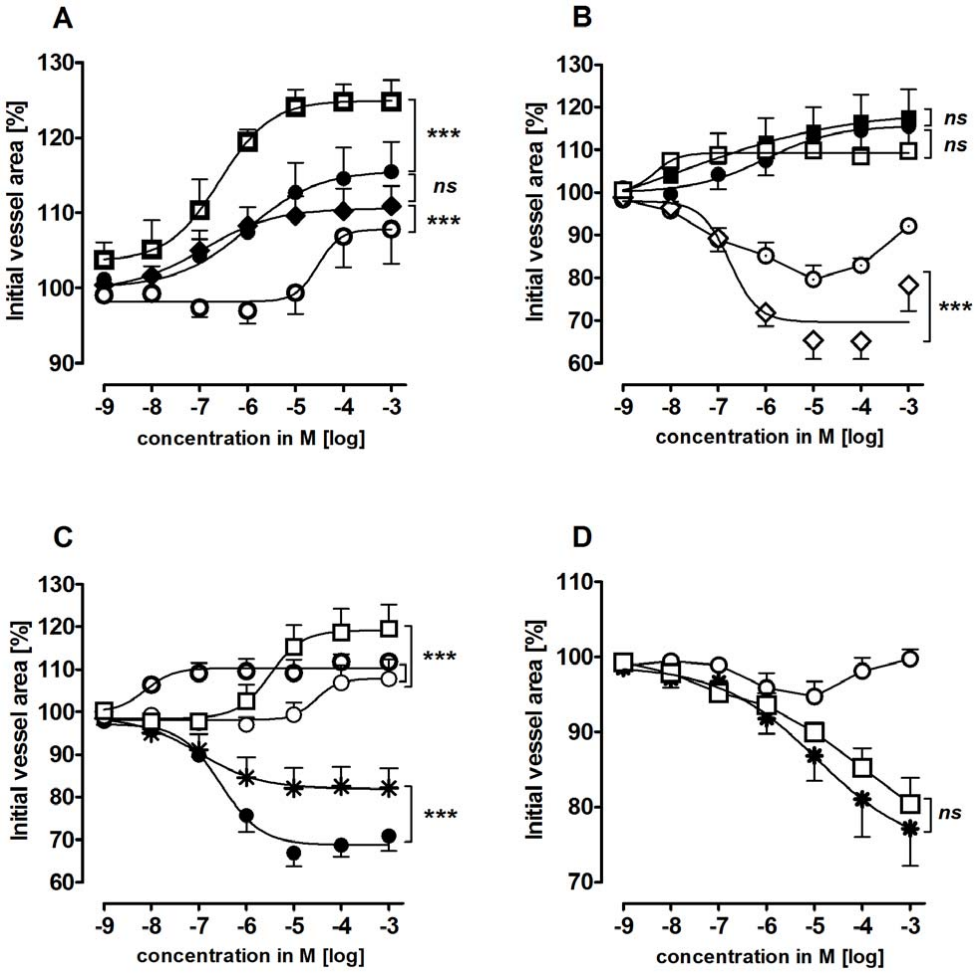
Responses of pulmonary veins (PV) to adrenergic agents. A) (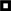) isoproterenol (n = 5); (•) epinephrine (n = 6); (⧫) procaterol (n = 7); (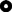) norepinephrine (n = 6); **B**) (•) epinephrine (n = 6); (▪) prazosine (100 nM), epinephrine (n = 6); (**
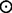
**) ICI 118551 (100 nM), epinephrine (n = 5); (**⋄**) ICI 118551 (10 µM), epinephrine (n = 5); (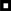) procaterol, epinephrine (n = 4); **C**) (○) norepinephrine (n = 6); (□) prazosine (100 nM), norepinephrine (n = 3); (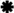) ICI 118551 (100 nM), norepinephrine (n = 9); (•) ICI 118551 (10 µM), norepinephrine (n = 6); (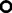) procaterol, norepinephrine (n = 4); **D**) (○) phenylephrine (n = 6); (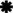) A 61603 (n = 5), (□) ICI 118551 (100 nM), phenylephrine (n = 5). Asterics indicate different EC_50_.values of the various curves. P<0.05 are considered as statistical significant and are indicated as followed 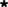 p<0.05, 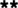 p<0.01 and 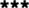 p<0.001.

In PVs, isoproterenol, procaterol, norepinephrine and epinephrine caused relaxation, with the following EC_50_ values: isoproterenol 0.26 µM, procaterol 0.12 µM, norepinephrine 30 µM and epinephrine 1 µM (Fig. 3A). Simultaneous treatment with epinephrine and the pure β_2_-agonist procaterol did not enhance epinephrine-induced relaxation (Fig. 3B). Further, combined treatment with norepinephrine and procaterol did not alter the maximal relaxant effect of norepinephrine; however EC_50_ values were shifted leftwards to lower concentrations, i.e. EC_50_ values were 8.6 nM for simultaneous treatment with procaterol and norepinephrine instead of 30 µM for norepinephrine alone. Pre-treatment with 100 nM prazosine had no effect alone (Table 1) or on epinephrine-induced relaxation (Fig. 3B), but enhanced the effect of norepinephrine (Fig. 3C). When PVs were pre-treated with the β_2_-antagonist ICI 118551 (10 µM or 100 nM) the effect of (nor)epinephrine was reversed and it became contractile (Fig. 3B/C). At 1 mM epinephrine showed some relaxation despite the presence of ICI 118551 (Fig. 3B). Similar results were obtained for (nor)epinephrine after pre-treatment with 1 µM propanolol (not shown). Neither ICI 118551 nor propanolol did affect the basal tone of PVs (Table 1). The α_1_-agonist A 61603 contracted PVs, whereas the α_1_-agonist phenylephrine had only a slight contractile effect at 10 µM that was reversed at higher concentrations (Fig. 3D). However, after pre-treatment with 100 nM ICI 118551 PVs responded to phenylephrine comparable to A 61603 (Fig. 3D). In addition, the α_2_-agonist clonidine, the β_1_-agonist denopamine and the β_3_-agonst CL 316243 had no effects in PVs (not shown).

### Effects of vasopressin on PAs and PVs

**Figure 4:**
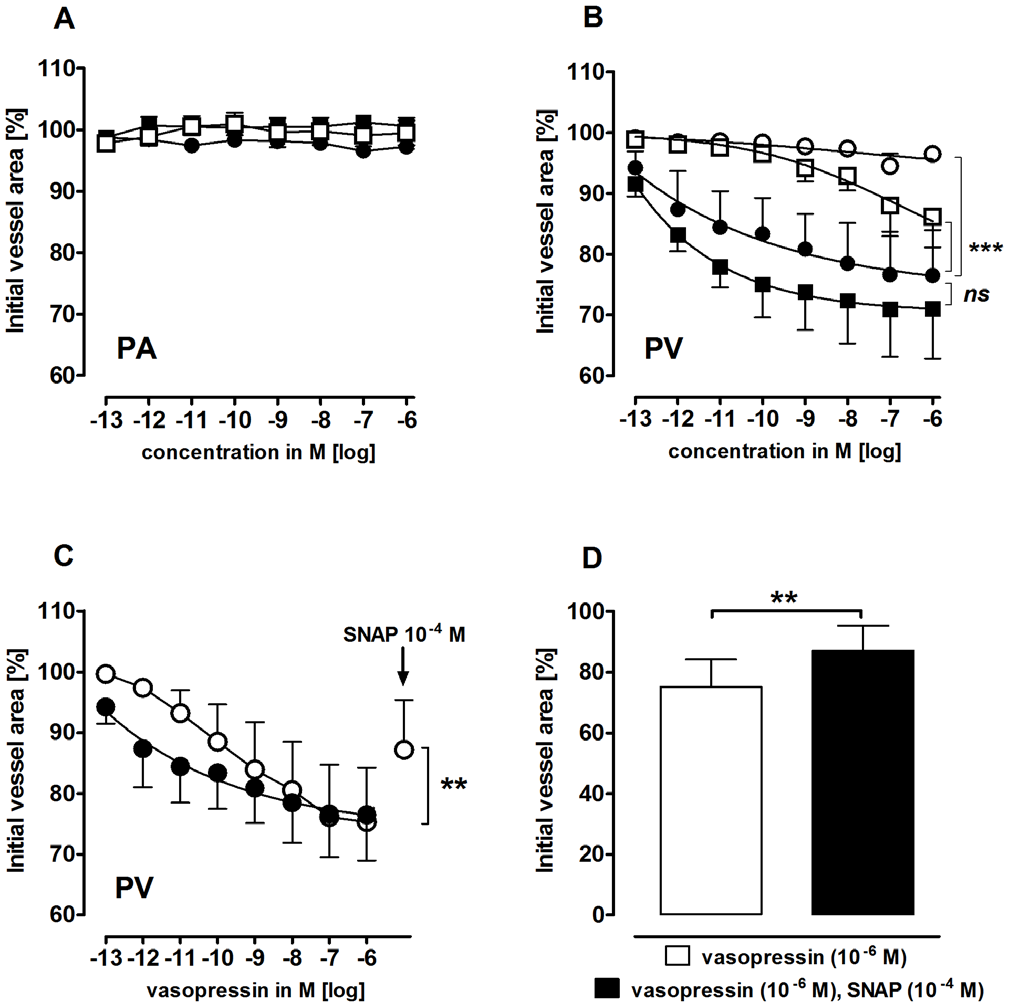
Responses of pulmonary arteries (PAs) and pulmonary veins (PVs) to vasopressin. **A**) PA: (•) vasopressin (n = 6); (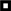) indomethacin (10 µM), vasopressin (n = 4); (▪) L-NAME (100 µM), vasopressin (n = 5) **B**) PV: (•) vasopressin (n = 5); (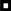) indomethacin (10 µM), vasopressin (n = 4); (▪) L-NAME (100 µM), vasopressin (n = 5); (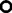) SR 49059 (10 nM), vasopressin (n = 3). **C**) PV: (•) vasopressin (n = 5); (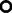) vasopressin, SNAP (n = 7). **D**) PV: vasopressin, SNAP (n = 7). **B**) Asterics indicate different EC_50_.values of the various curves. **C–D**) Statistics was performed using the Wilcoxon test. For all: P<0.05 are considered as statistical significant and are indicated as followed 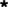 p<0.05, 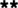 p<0.01 and 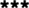 p<0.001.

**Figure 5:**
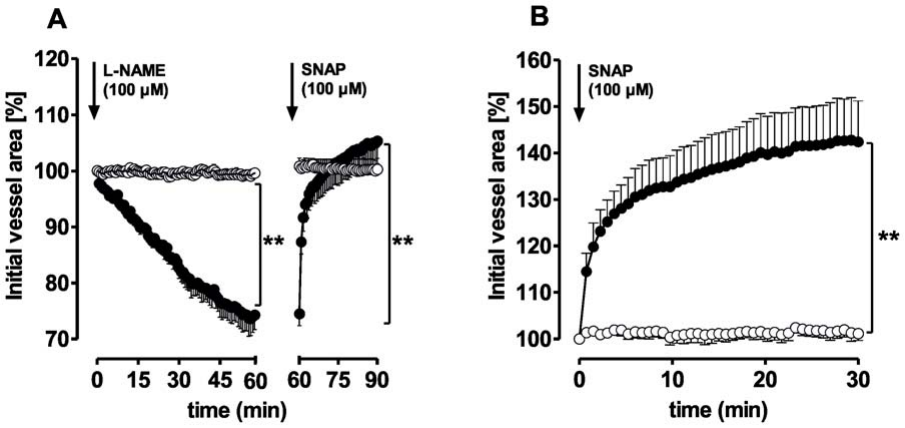
Responses of pulmonary arteries (PAs) and pulmonary veins (PVs) to L-NAME and SNAP. **A**) (•) PV (n = 3); (○) PA (n = 3). **B**) (•) PV (n = 3); (○) PA (n = 3). Statistics was conducted using a linear mixed model analysis. P-values were adjusted for multiple comparisons by the false discovery rate. P<0.05 are considered as statistical significant and indicated as followed 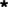 p<0.05, 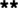 p<0.01 and 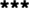 p<0.001.

The neurohypophyseal peptide vasopressin contracts vascular smooth muscles via binding to V_1a_ receptors. PVs, but not PAs, contracted to vasopressin (EC_50_: 2 pM, Fig. 4A/B). The cyclooxygenase inhibitor indomethacin (10 µM) attenuated vasopressin-induced contraction. Conversely, the NO synthase inhibitor N-nitro-L-arginine methyl ester (L-NAME, 100 µM) showed a trend towards enhancement (Fig. 4B). However, neither L-NAME nor indomethacin changed the vascular responses of PAs to vasopressin (Fig. 4A). Pre-treatment of PVs with the V_1a_ antagonist SR 49059 (10 nM) showed no effect alone (Table 1), but abolished contraction due to vasopressin (Fig. 4B). Further, the NO-donor S-Nitroso-N-acetyl-DL-penicillamine (SNAP) partly reversed vasopressin-induced contraction in PVs (Fig. 4B). Indomethacin slightly contracted PVs (Table 1) and L-NAME induced time-dependent contraction (Fig. 5A), which was reversed by SNAP. Further, native PVs also relaxed to SNAP (Fig. 5B), whereas PAs did not react to L-NAME or SNAP (Fig. 5A/B).

### Effects of angiotensin II on PAs and PVs

**Figure 6:**
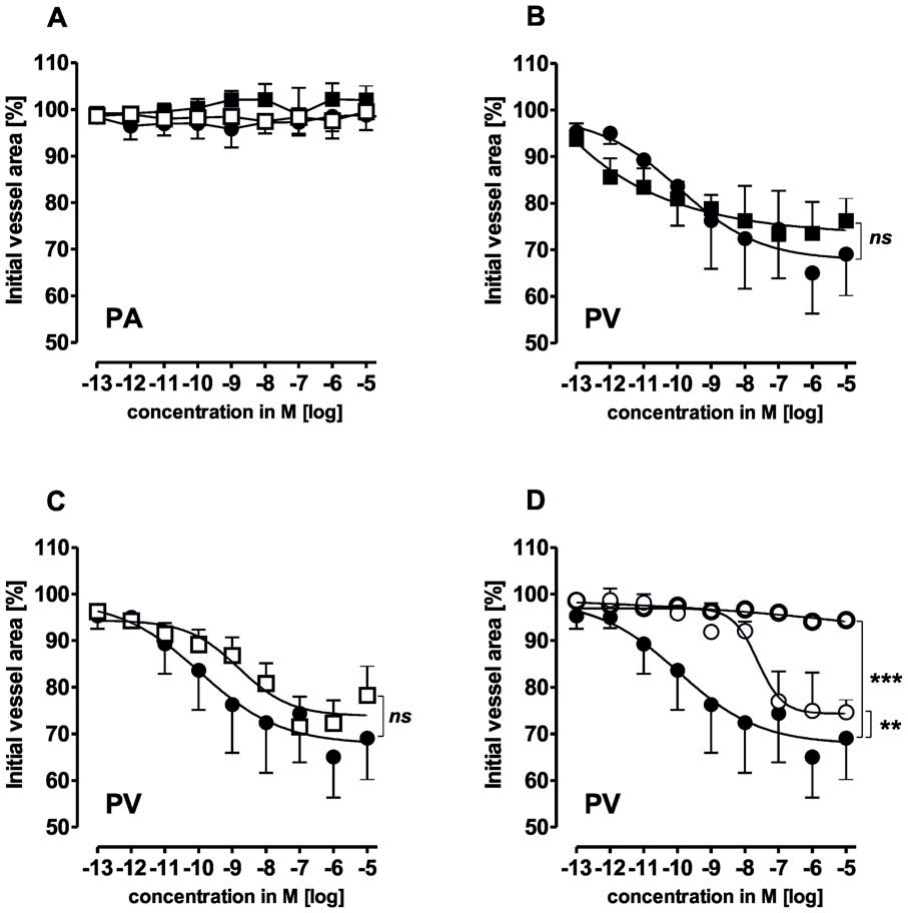
Responses of pulmonary arteries (PAs) and pulmonary veins (PVs) to angiotensin II. **A**) PA: (•) angiotensin II (n = 6); (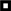) indomethacin (10 µM), angiotensin II (n = 5); (▪) L-NAME (100 µM), angiotensin II (n = 5) **B**) PV: (•) angiotensin II (n = 5); (▪) L-NAME (100 µM), angiotensin II (n = 5) **C**) PV: (•) angiotensin II (n = 5); (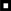) indomethacin (10 µM), angiotensin II (n = 5) **D**) PV: (•) angiotensin II (n = 5); (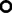) losartan (1 µM), angiotensin II (n = 3); (○) losartan (10 nM), angiotensin II (n = 3). Asterics indicate different EC_50_.values of the various curves. P<0.05 are considered as statistical significant and are indicated as followed 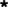 p<0.05, 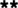 p<0.01 and 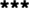 p<0.001.

Angiotensin II induces vasoconstriction via the G-protein coupled AT_1_ receptor. Angiotensin II contracted PVs (EC_50_: 0.1 nM; Fig. 6B–D), but not PAs (Fig. 6A). Pre-treatment with indomethacin (10 µM) or L-NAME (100 µM) did not alter the response of pulmonary vessels to angiotensin II (Fig. 6A–C). In order to analyse, whether angiotensin-induced contraction is specific to AT_1_ binding, PCLS were pre-treated with the AT_1_ antagonist losartan. Losartan (1 µM) had no effect alone, but abolished the effect of angiotensin II (Fig. 6D, Table 1).

## Discussion

(Nor)epinephrine and vasopressin are clinically relevant cardiovascular agents that are daily applied in the treatment of acute haemodynamic instability. Further, angiotensin-converting enzyme inhibitors and AT_1_ antagonists are used to treat chronic heart failure. Commonly, their effects on systemic circulation are well controlled; whereas their pulmonary vascular effects are rarely assessed, especially in PVs. However this entity is of relevance, especially in pulmonary hypertension and right heart failure. This study compared the pulmonary vascular effects of (nor)epinephrine, vasopressin and angiotensin II in PCLS of GPs. Adrenergic agonists contracted PAs and PVs, but their contractile effect on PVs was unmasked only in the presence of β_2_-inhibition. Vasopressin and angiotensin II only contracted PVs.

### Alpha - and β-adrenergic stimulation

(Nor)epinephrine interacted with α_1_/β_1/2_-adrenoceptors of PAs and PVs. (Nor)epinephrine-related contraction of PAs was inhibited by prazosine and thus most likely caused by activation of α_1_-adrenoceptors. In addition, inhibition of ß_1_-adrenoceptors abolished epinephrine-induced contraction, suggesting their involvement in α_1_-mediated contraction. This somewhat surprising conclusion is supported by two findings: First, α_1_/β_1/2_-agonists such as (nor)epinephrine contracted PAs stronger than phenylephrine and A 61603, which selectively act on α_1_-adrenoceptors in PAs. Second, the selective β_2_-agonist procaterol relaxed PAs, whereas the β_1/2_-agonist isoproterenol did not, suggesting neutralization of β_1_-mediated contraction by β_2_-mediated relaxation. Concurrent activation of α_1_/β_1_-adrenoceptors was before reported in PVs [11], albeit with regard to ectopic activity. Previously, isoproterenol that binds stronger on β_1_-adrenoceptors than on β_2_-adrenoceptors [12] was observed to contract vessels [13]. Of note, the β_1_-agonist denopamine failed to contract PAs or to enhance phenylephrine-induced contraction. These findings suggest a complex and indirect activation of β_1_-adrenoceptors by epinephrine that remains to be further elucidated.

Interestingly, the contractile potency of epinephrine decreased above 1 µM indicating the additional activation of β_2_-adrenoceptors and subsequent vasorelaxation. This conclusion is supported by the effects of procaterol that relaxed PAs and prevented epinephrine- and norepinephrine-induced contraction. Further, treatment of PAs with ICI 118551 enhanced norepinephrine-related contraction. Thus, vasoconstriction appears to be partially masked by stimulation of β_2_-adrenoceptors. In contrast, ICI 118551 did not alter epinephrine-induced contraction. Possibly, epinephrine at 1 µM mainly acts as a relatively pure α_1_-agonist, whereas above 1 µM it competes with ICI 118551 for β_2_-adrenoceptors.

Thus, adrenergic agents interact in a complex manner with α_1_/β_1/2_-adrenoceptors of PAs (Table 2). Contraction is mainly mediated by α_1_-adrenoceptors, but is antagonised by β_2_-adrenoceptors and aggravated by β_1_-adrenoceptors.

**Table 2:** Overview of cardiovascular agents and their receptor-mediated vascular effects.

agents	PA/contraction	PA/relaxation	PV/contraction	PV/relaxation
**epinephrine 1 µM**	α_1_ (ß_1_)		(α_1_)	β_2_
**norepinephrine 1 µM**	α_1_		(α_1_)	β_2_
**epinephrine 1 mM**	α_1_	β_2_	(α_1_)	β_2_
**norepinephrine 1 mM**	(α_1_)	(β_2_)	(α_1_)	β_2_
**phenylephrine**	α_1_		(α_1_)	(β_2_)
**A 61603**	α_1_		α_1_	
**procaterol**	–	β_2_	–	β_2_
**>1 pM vasopressin**	–	–	V_1a_	–
**angiotensin II**	–	–	AT_1_	–
**NO**	–	–	–	NO

Masked effects are expressed with ().

The pulmonary venous vascular bed contributes up to 40% to total pulmonary vascular resistance [6]. In PVs, β_2_-adrenoceptors appear to be dominant and mask the activation of α_1_-adrenoceptors (Table 2). This is concluded from the observation that PVs contracted to (nor)epinephrine and phenylephrine only in the presence of β_2_-blockers. Given alone, (nor)epinephrine was relaxant, whereas phenylephrine was not, which is in line with its weak β_2_-affinity. Pre-treatment with prazosine only enhanced the relaxant effect of norepinephrine. Probably, maximal relaxation of epinephrine was already reached, as it stimulates β_2_-adrenoceptors more potently than norepinephrine [12]. Further, combined treatment with procaterol and epinephrine did not alter epinephrine-induced relaxation in PVs, probably due to similar binding affinities in respect of the β_2_-receptor [14]. However, as expected, simultaneous treatment with procaterol and norepinephrine was superior compared to norepinephrine alone and is in line with the high binding affinity of procaterol to the β_2_-receptor [14].

Our results may help to put previous findings into perspective. In perfused feline lung lobes, norepinephrine and phenylephrine enhanced pulmonary vascular resistance; while epinephrine and isoproterenol had the opposite effect [15]. These results might reflect the net effect of α_1_-dependent pulmonary arterial contraction versus β_2_-dependent pulmonary venous relaxation. Though, in perfused rat lungs norepinephrine and phenylephrine reduced the pulmonary perfusion pressure [5], likely due to β_2_-mediated relaxation [16].

Beta_2_-mediated pulmonary venous relaxation is of clinical interest, as mixed β_1/2_-blockers are widely-used. Commonly, α_1_/ß_1/2_-agonists, such as (nor)epinephrine are applied in heart failure or shock. However, if these patients are pre-treated with mixed β_1/2_-blockers, circulation support with (nor)epinephrine might worsen gas exchange, due to pulmonary venoconstriction, as (nor)epinephrine mainly activate α_1_-adrenoceptors, while ß_2_-adrenoceptors are still blocked. In pulmonary hypertension, (nor)epinephrine might increase right ventricular afterload and aggravate right ventricular failure. If these findings could be confirmed in humans, this should be considered in the therapy of heart failure.

### Vasopressin

Vasopressin has various physiological functions, including V_1a_ receptor-mediated regulation of blood pressure and V_2_ receptor-mediated control of body water [17]. Further, its relevance in resuscitation is increasingly discussed. Here, vasopressin only contracted PVs. Inhibition of endothelial NO-synthase (eNOS) tended to enhance this contractile effect and the NO-donor SNAP reversed it in part. Further, L-NAME given alone also contracted PVs. This indicates the critical role of eNOS in PVs as opposed to arteries, similar to observations in human [18] and porcine PVs [19]. Moreover, indomethacin attenuated the effect of vasopressin, indicating its partial action through the release of contractile prostanoids such as thromboxane.

In contrast to our results, vasopressin relaxed PAs in isolated perfused rat lungs [3] and isolated canine pulmonary vessels in dependence to eNOS [1], [4]. In dogs, it contracted PAs [2]. Vasopressin contracts vessels via V_1a_ receptor-mediated phospolipase C activation and IP_3_-signalling [17] and opposing to our results, the involvement of relaxant prostaglandins was shown [20]. Thus, the effect of vasopressin in pulmonary vessels may strongly depend on the studied species [21]. Best to our knowledge, pulmonary venous contraction due to vasopressin was not yet reported.

Interestingly, human data indirectly support our results [22]: application of vasopressin led to enhanced pulmonary capillary wedge pressures (PCWP). Vice versa, application of the V_1a/2_ antagonist conivaptan decreased PCWP [23], whereas the V_2_ antagonist tolvaptan did not, but increased vasopressin plasma levels [24]. Thus, vasopressin antagonists that do not block V_1a_ receptors might be problematic. In patients with heart failure, vasopressin plasma levels are increased up to 0.28 nM [25], concentrations that contracted PVs in our *in vitro* model. Hence, vasopressin-related contraction of PVs might enhance pulmonary hydrostatic pressures, left ventricular preload and wall stress. Taken together, our findings suggest that V_1a_ antagonists might reduce pulmonary complications in heart failure and further, that vasopressin might not worsen right ventricular afterload (Table 2).

### Angiotensin II

Angiotensin II is the key peptide of the renin-angiotensin system and mainly produced in the pulmonary arterial vascular bed [26]. In the present study, angiotensin II only contracted the PVs; neither inhibition of NO- nor prostanoid synthesis altered this response.

In contrast to our results, angiotensin contracted endothelium denuded, isolated PAs of GPs and dogs [4], [27], [28], whereas canine PVs relaxed [4] or failed to respond [28]. Moreover, indomethacin enhanced the contractile effect of angiotensin in PAs [4]. In line with our results, angiotensin contracted rat PVs [29]. Further, in patients with atrial and ventricular septum defects, angiotensin increased left atrial and pulmonary venous pressures, but did not alter the pulmonary arterial resistance [30]. In addition, only the extent of left to right shunts increased. According to our results, pulmonary venoconstriction might be a reasonable explanation for these observations. Prevention of pulmonary venous contraction by AT_1_ antagonists or ACE-inhibitors might contribute to their beneficial effect in heart failure.

### PCLS from GPs

Thus, our findings are in line with clinical studies and suggest that PCLS from GPs resemble human pulmonary vascular pharmacology reasonably well, as already indicated for airway pharmacology [9]. This study was performed *in vitro* and thus excludes factors that affect vascular responses *in vivo* such as shear stress or embolism. *In vivo,* the PA can be accessed by catheterization, whereas the access of PVs is more difficult. The PCWP relates to left atrial pressure and to large PVs, but small PVs are not reflected [31]. Further, vascular pressure represents a product of vascular tone and filling. Hence, it is influenced by ventricular contractility. Complementary to *in vivo* studies, PCLS allow exclusively studying the vascular tone of pulmonary vessels.

For the first time, this study systematically compared the effects of clinically relevant cardiovascular agents simultaneously on PAs and PVs. Our results indicate that PAs and PVs are contracted by α_1_-agonists, while relaxation, which occurs predominantly in PVs, is mediated by β_2_-adrenoceptors. Of note, β_1_-adrenoceptors contribute to adrenergic contraction in PAs. Further, vasopressin and angiotensin target predominantly PVs and thus raise the hypothesis that activation of V_1a_ receptors and AT_1_ receptors favours pulmonary oedema. Thus, clinically, the application of vasopressin in left heart failure should be faced with caution and conversely suggests a beneficial role of V_1a_ and AT_1_ antagonists. In contrast to vasopressin, (nor)epinephrine may increase right ventricular afterload, but not pulmonary oedema, except in patients pre-treated with β_1/2_-inhibitors. Taken together, both vascular beds exhibit important differences in their responses to cardiovascular drugs. In conclusion, successful restoration of circulation in heart failure and shock should take into account the differential effects of cardiovascular agents on PAs and PVs.
